# Supplementary material for: Computationally efficient design of directionally compliant metamaterials
Source: Nat Commun. 2019 Jan 17;10:291. doi: 10.1038/s41467-018-08049-1 (PMC6336888; doi:10.1038/s41467-018-08049-1)
Supplement: Supplementary file 1 — Supplementary Information [file 41467_2018_8049_MOESM1_ESM.pdf]

Computationally Efficient Design of Directionally Compliant Metamaterials

Shaw et al.

Supplementary Figures

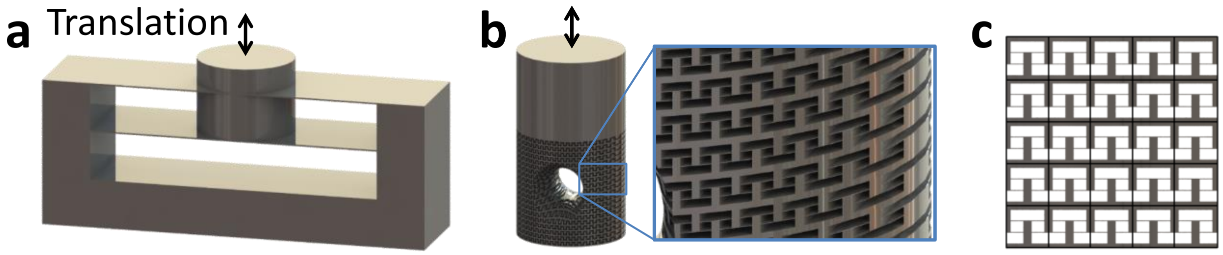

Supplementary Figure 1. Directionally compliant metamaterials (DCMs) as irregularly shaped flexure systems. **a**, A flexure system that achieves a compliant translation. **b**, An irregularly shaped DCM that achieves the same compliant translation, and **c**, a close up of its periodic lattice of repeating cells.

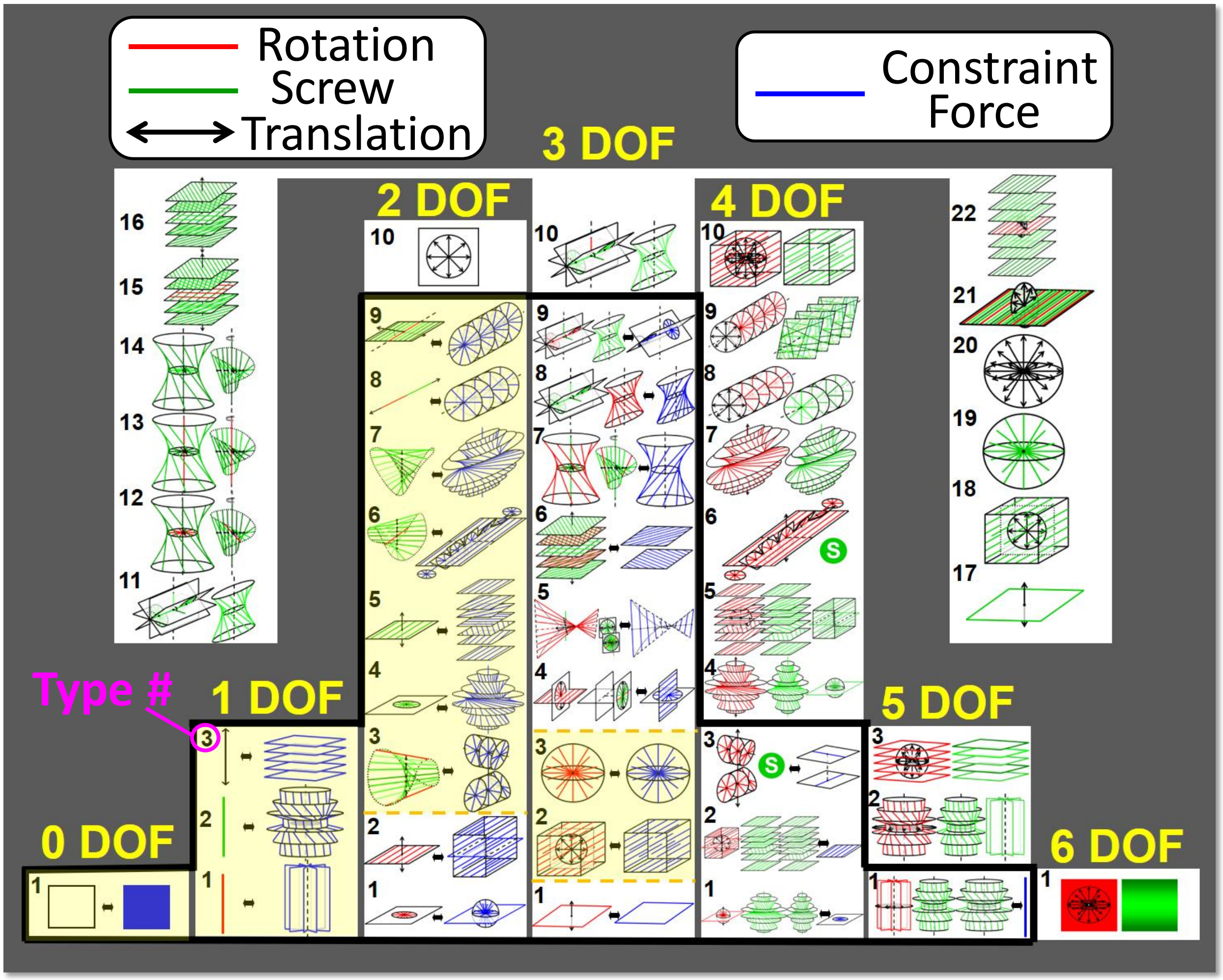

Supplementary Figure 2. "Freedom and constraint topologies" (FACT) library of vector spaces. All 50 freedom spaces are provided within 7 columns organized according to the number of degrees of freedom (DOFs) that constitute the freedom spaces in each column. The freedom spaces within the black-outlined pyramid link to constraint spaces that possess enough pure-force wrench vectors (PFWVs) to synthesize parallel systems. The constraint spaces within the region shaded yellow, called cell spaces, are the only spaces that can be used to synthesize directionally compliant metamaterial (DCM) cells.

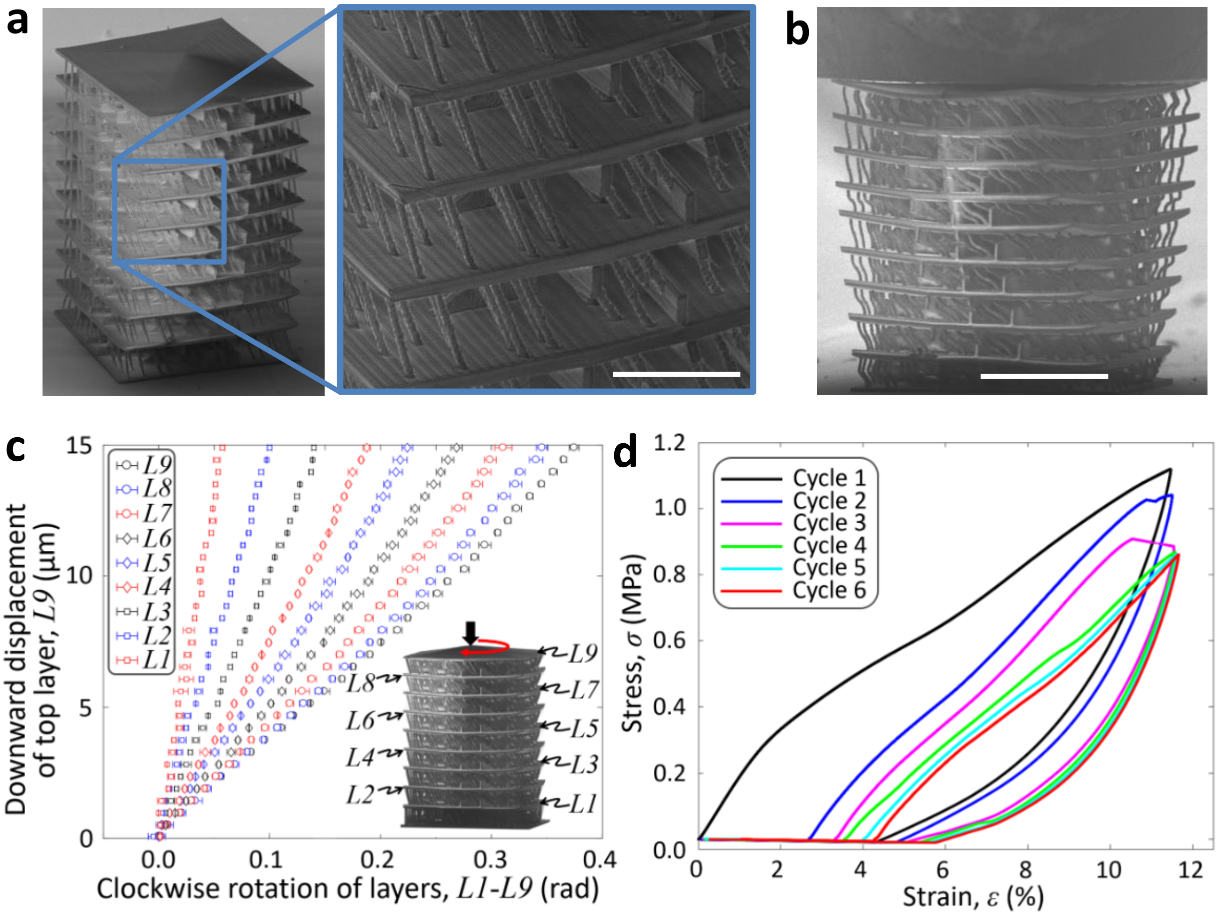

Supplementary Figure 3. Additional screw directionally compliant metamaterial (DCM) results. **a**, DCM fabricated using two-photon lithography. **b**, DCM compression test. **c**, Displacement of the DCM's top layer versus the rotation of each layer. The experimental data points correspond to the averaged rotation of each layer for three identical samples, while the error bars correspond to the standard deviation amongst the samples. **d**, Stress versus strain plotted for 6 cycles (scale bar in **a**, 20 μm, **b**, 50 μm).

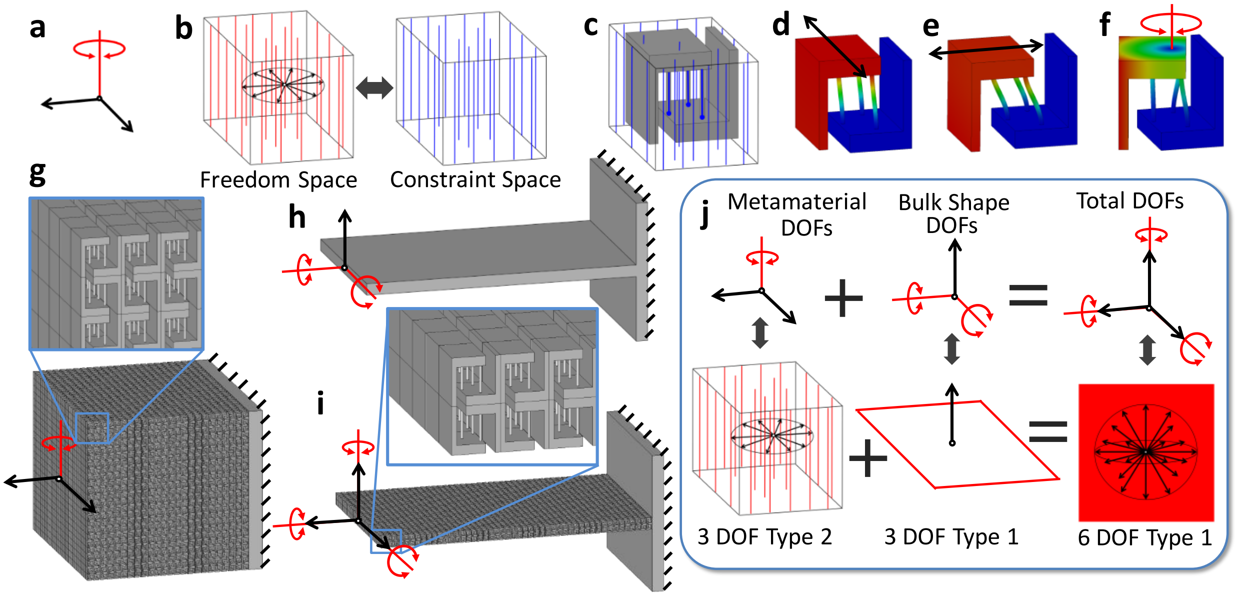

Supplementary Figure 4. Directionally compliant metamaterial (DCM) shape affects degrees of freedom (DOFs). a, Three DOFs; **b**, their freedom space and complementary constraint space, **c**, used to synthesize each cell such that they, **d-f**, achieve the DOFs within, **g**, a cube-shaped DCM. **h**, A blade-shaped homogenous material achieves three different DOFs. **i**, A blade-shaped DCM with the architecture of **g** achieves 6 DOFs. **j**, The freedom space of a DCM results from the sum of its architecture's freedom space and its bulk shape's freedom space (colors in **d-f** are defined in Fig. 3b).

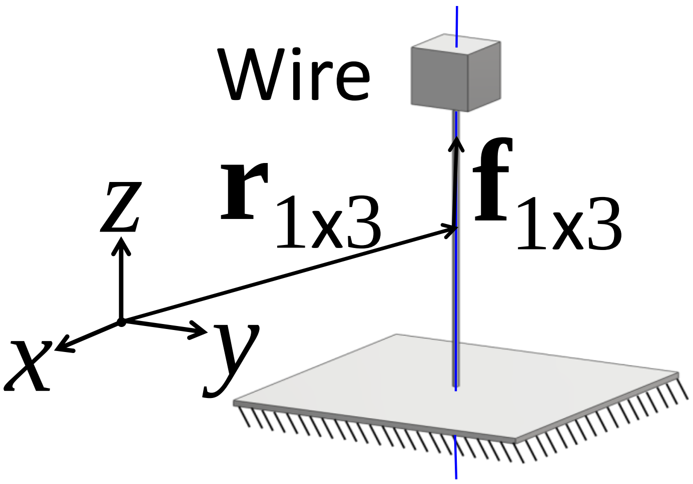

Supplementary Figure 5. Pure-force wrench vector (PFWV) parameters and wire element. The parameters necessary to mathematically define a PFWV, which can be used to model the behavior of a simple wire element.

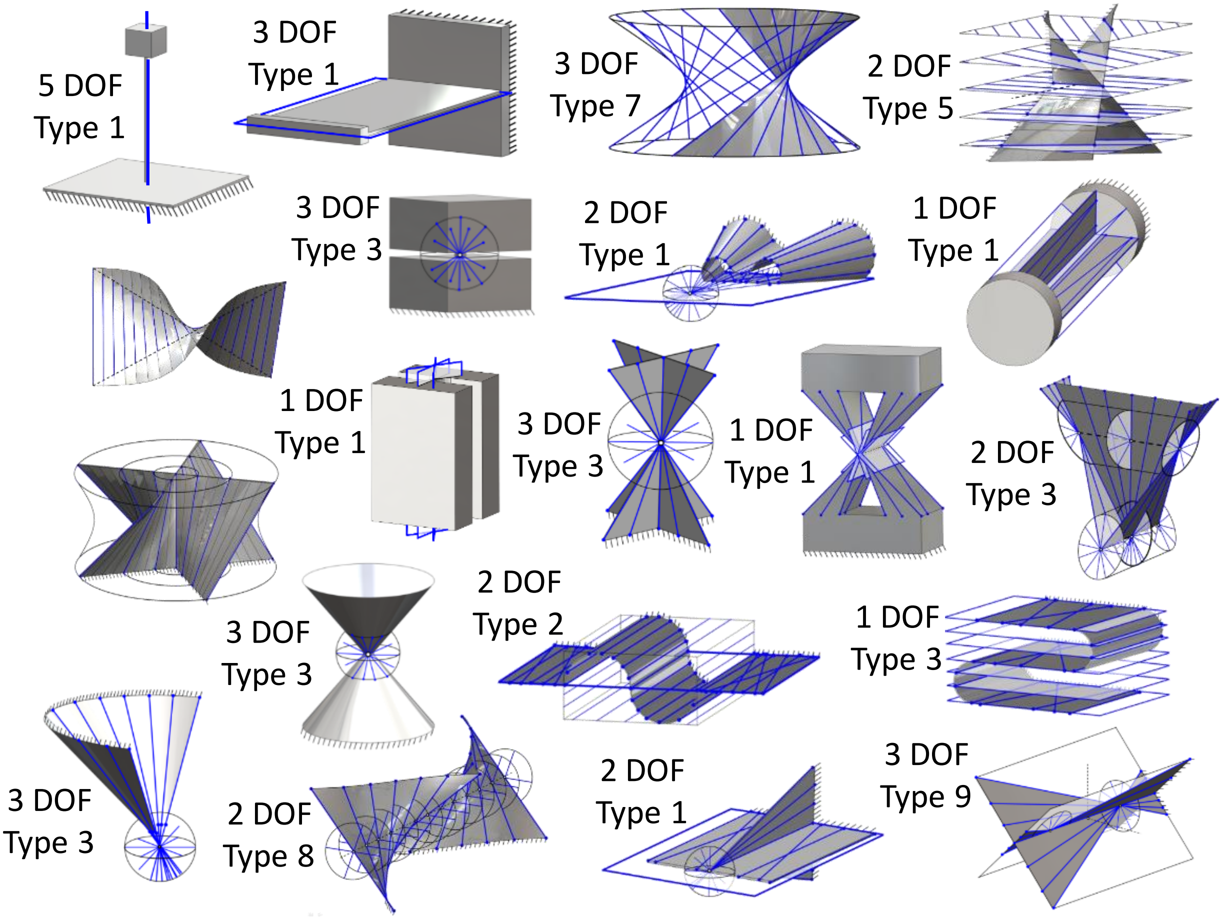

Supplementary Figure 6. Example flexible elements. A variety of flexible element geometries shown with their corresponding constraint spaces labeled according to the convention established in the freedom and constraint topologies (FACT) library of Supplementary Fig. 2.

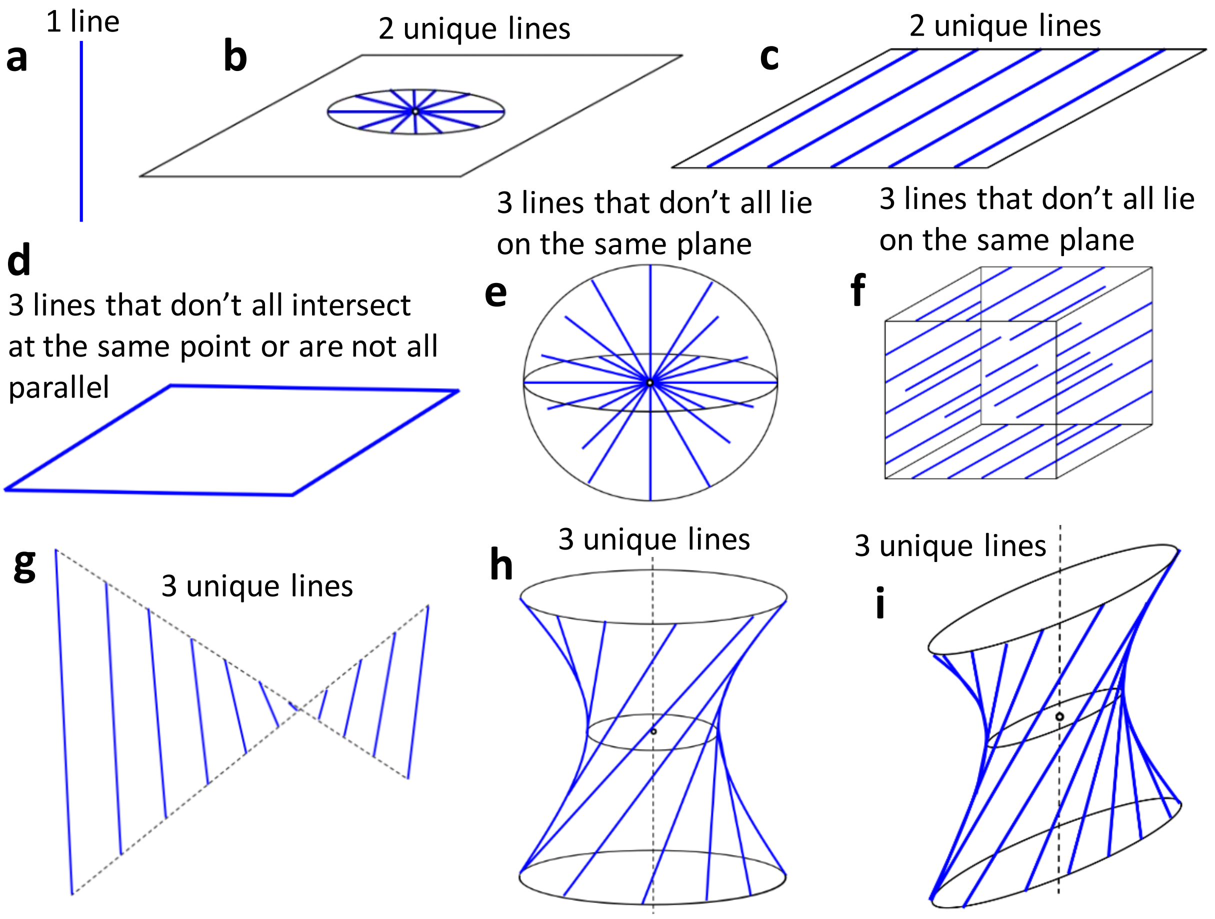

Supplementary Figure 7. Rules for selecting independent pure-force wrench vectors (PFWVs) from within the 9 basic shapes that constitute all constraint spaces. a, Single line. **b**, Disk of lines. **c**, Plane of parallel lines. **d**, Plane of lines. **e**, Sphere of lines. **f**, Box of parallel lines. **g**, Hyperbolic paraboloid of lines. **h**, Circular hyperboloid of lines. **i**, Elliptical hyperboloid of lines.

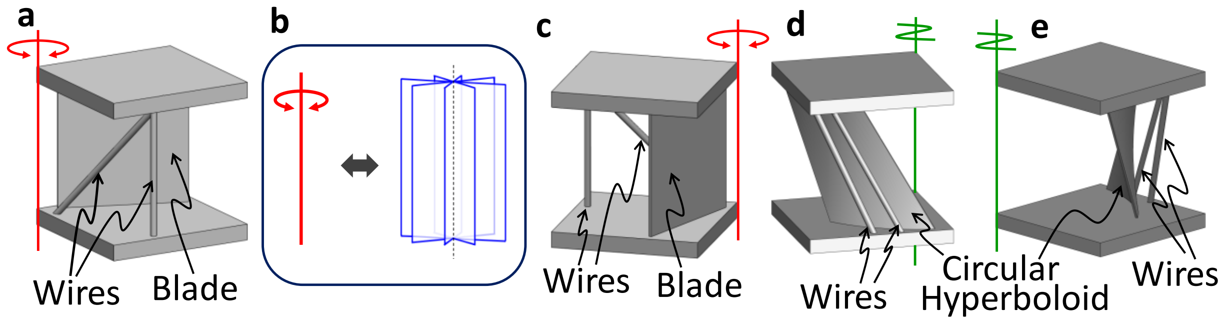

Supplementary Figure 8. Example exactly-constrained cells synthesized using different flexible elements. a, A cell that achieves a single rotational degree of freedom (DOF) and consists of a blade and two wire elements. **b**, The cell's freedom and constraint spaces. **c**, A different view of the same cell. **d**, **e**, Two different views of a cell that achieves a single screw DOF and consists of a circular hyperboloid element and two wire elements.

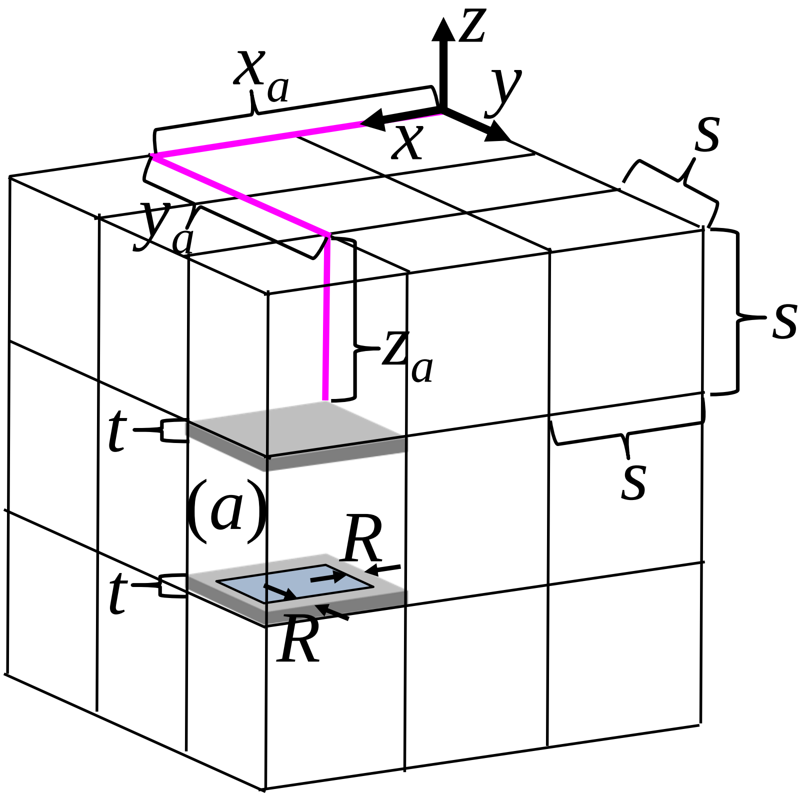

Supplementary Figure 9. Parameters used within the automated design tool. The tool synthesizes each cell within a directionally compliant metamaterial (DCM) with the minimum necessary number of independent wire elements that lie within the DCM's desired constraint space and directly join the cell's two rigid bodies together. The two bodies of cell (*a*) are shown with corresponding parameters.

Supplementary Note 1: Additional applications for directionally compliant metamaterials (DCMs)

In addition to enabling soft-robotic joints (Fig. 1b) that achieve directionally compliant properties and bodies that passively deform in controlled ways such as the propeller example with the screw degree of freedom (DOF) (Fig. 2c) discussed in the main text, DCMs can be used to enable new precision flexure systems with demanding shape requirements. Flexure systems currently achieve compliant directions according to how they are shaped on the macroscale. The flexure system of Supplementary Fig. 1a, for instance, achieves a single translational DOF with high compliance because its homogenous constituent material is shaped with macro-sized parallel blade elements (see Supplementary Movie 1). Suppose, however, a system is desired that achieves the same translational DOF but must fit within a cylindrical shape with a filleted circular hole through its geometry. Such a system would not be possible to achieve by shaping a homogenous material as a macroscale flexure system but would necessitate the implementation of a DCM like the example in Supplementary Fig. 1b. Although the DCM of Supplementary Fig. 1b consists of a single repeating cell design (Supplementary Fig. 1c), most other DCMs typically require aperiodic cell designs, which differ throughout the material's geometry. Since the theory of this paper enables the design of any kind of DCM including aperiodic designs, this theory will disrupt the field of flexure-system design such that flexure systems will be enabled that can conform to almost any desired shape.

Supplementary Note 2: Example that demonstrates the effect of a DCM's bulk shape

This section provides a supplementary example that demonstrates how the DOFs achieved by a DCM are similarly affected by both the DCM's architecture and its bulk shape. Supplementary Movie 5 provides animations of the example presented here. Suppose a cube-shaped DCM is desired that achieves two orthogonal translations and an orthogonal rotation as shown in Supplementary Fig. 4a. These DOFs combine to generate the 3 DOF Type 2 freedom space in Supplementary Fig. 2. This freedom space consists of an infinitely large box of rotation lines that are parallel to the axis of the rotational DOF and a disk of translation arrows that are orthogonal to this axis as shown in Supplementary Fig. 4b. The freedom space's complementary constraint space is an infinitely large box of pure-force wrench vectors (PFWVs) that are parallel to the rotation lines of the freedom space. Since the freedom space contains n=3 DOFs, three wire elements should be selected according to equation (4) with axes that are colinear with m=3 independent PFWVs from the constraint space (Supplementary Fig. 4c) so that the resulting cell achieves its desired DOFs (Supplementary Fig. 4d-f). If this process is repeated for all the cells within the cube-shaped DCM, a periodic design can be generated that achieves the desired DOFs (Supplementary Fig. 4g). Suppose, however, that the DCM is shaped like a flat blade instead of a cube. Whereas a cube-shaped homogenous material would achieve no DOFs, a blade-shaped homogenous material would achieve the two orthogonal intersecting rotations and the orthogonal translation shown in Supplementary Fig. 4h. If the same architecture shown in Supplementary Fig. 4g where applied to a blade-shaped DCM (Supplementary Fig. 4i), the resulting material would exhibit 6 DOFs. Thus, the freedom space of a DCM is determined by linearly combining the twist

vectors that constitute (i) the freedom space of the DCM's architecture and (ii) the freedom space of the DCM's bulk shape if it were filled with a homogenous material (Supplementary Fig. 4j).
