# Supplementary material for: Computationally efficient design of directionally compliant metamaterials
Source: Nat Commun. 2019 Jan 17;10:291. doi: 10.1038/s41467-018-08049-1 (PMC6336888; doi:10.1038/s41467-018-08049-1)
Supplement: Supplementary file 3 — Description of Additional Supplementary Files [file 41467_2018_8049_MOESM3_ESM.pdf]

Description of Additional Supplementary Files

File Name: Supplementary Movie 1
Description: This movie contains animated examples of directionally compliant metamaterial (DCM) applications that require demanding shape requirements.

File Name: Supplementary Movie 2
Description: This movie animates the steps of the freedom and constraint topologies (FACT) approach necessary to design a directionally compliant metamaterial (DCM) that achieves a single screw degree of freedom (DOF). It also provides a video of an in situ scanning electron microscope (SEM) compression test performed on a microfabricated version of the screw DCM designed. 3

File Name: Supplementary Movie 3
Description: This movie animates the steps of the freedom and constraint topologies (FACT) approach necessary to design three directionally compliant metamaterial (DCM) examples—a single degree-of-freedom (DOF) example, a multi-DOF example, and a multi-DOF example with a freedom space that links to a constraint space that is a cell space.

File Name: Supplementary Movie 4
Description: This movie provides a real-time demo of the automated design tool provided in Supplementary Software. It also provides animations of complex directionally compliant metamaterial (DCM) designs generated using the tool.

File Name: Supplementary Movie 5
Description: This movie provides examples that demonstrate the principle that a directionally compliant metamaterial's (DCM's) bulk shape and architecture equally affect its DOFs.

File Name: Supplementary Software
Description: The automated design tool consists of three MATLAB files. The master file, MaterialDesignGUI.m, contains the design tool's functions. The file, MaterialDesignGUI.fig, contains the content necessary to launch the graphical user interface (GUI). The file, images.mat, contains various images required for the GUI. Please click on the following link or paste the universal resource locator (URL) directly into a browser to download the three required files. Once they have been downloaded, put them all into the same folder on your computer and click on the MaterialDesignGUI.m file to open it with MATLAB. Once the file is open, run it to launch the tool. [https://github.com/jonathanbhopkins/Computationally-Efficient-Design-of-Directionally-Compliant-Metamaterials.git]
